# Two Clinical Records of Human Injuries with a Death caused by Electric Eels *Electrophorus* spp. Gill, 1864

**DOI:** 10.1590/0037-8682-0007-2025

**Published:** 2025-07-07

**Authors:** Vidal Haddad, Flávio César Thadeo de Lima, William Gareth Richard Crampton, Ribamar Ponsiano Monteiro, Raimundo Nonato Gomes Mendes-Júnior

**Affiliations:** 1 Universidade Estadual Paulista, Faculdade de Medicina de Botucatu, Departamento de Dermatologia e Infectologia, Botucatu, SP, Brasil.; 2 Universidade Estadual de Campinas, Instituto de Biologia, Campinas, SP, Brasil.; 3 University of Central Florida, Department of Biology, 4110 Libra Drive, Orlando, Florida, 32816, USA.; 4 Instituto Guarda-Parque do Jari, Laranjal do Jari, AP, Brasil.; 5 Museu de Zoologia da Universidade de São Paulo, Programa de Pós-Graduação em Sistemática, Taxonomia Animal e Biodiversidade, São Paulo, SP, Brasil.; 6 Instituto Chico Mendes de Conservação da Biodiversidade, Reserva Extrativista do Rio Cajari, Macapá, AP, Brasil.

**Keywords:** Poraqué, Temblón, Electric Shock, Drowning

## Abstract

Electrical eels, or poraqués or temblones (*Electrophorus* spp. Gill, 1864), are highly feared animals in the Amazon, mainly because of their electric discharge of up to 860 volts. We report two cases of human injuries caused by electric eels, one of which was fatal. The shocks caused muscle rigidity, and the victim likely drowned because of muscle contracture. Strong electric shocks increase the levels of cardiac enzymes in the blood, as observed with the elevation of total creatine phosphokinase (CPK) and creatine phosphokinase myocardial band (CPK-MB) in surviving victims, which is potentially indicative of injuries caused by poraqués.

## INTRODUCTION

**FIGURE 1:**
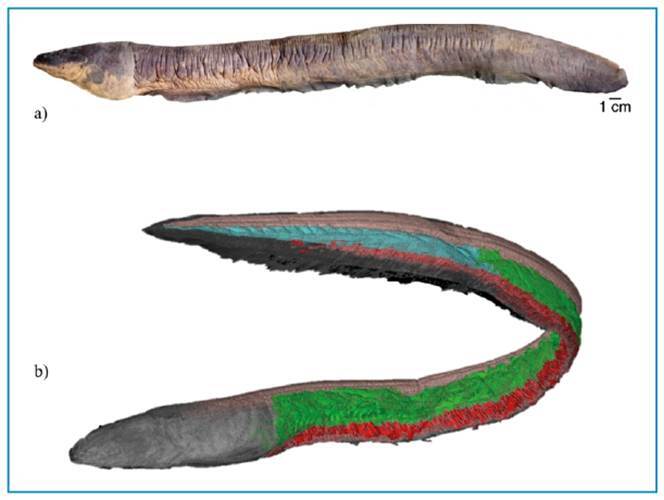
**a)** *Electrophorus voltai* holotype, MPEG 15529, from de Santana *et al.* 2019. b) Iodine contrast microCT of *Electrophorus voltai* MZUSP 127731’s electric organs: Main organ (green), Sach´s organ (blue) and Hunter´s organ (red). The postvisceral *epaxialis* muscles are shown (pink).

Freshwater ecosystems host diverse animals capable of causing injury through bites, stings, or toxin inoculation^1^ ^,^ ^2^. In Brazil, the highest incidence of injury from freshwater animals occur in the Amazon region^1^ ^-^ ^3^, including for electric eels^4^. Electric eels (*Electrophorus* spp.), the poraqués or temblones, are among the most feared animals in the Amazon^3^ ^-^ ^5^. They are large (up to 2m) eel-like fish belonging to the order Gymnotiformes, which also comprises 270 species of weakly electric knifefishes^3^ ^-^ ^6^. All gymnotiform fish generate electric organ discharges (EODs), which reach no more than ~1V in knifefishes, but up to 860V in electric eels^5^ ^,^ ^6^ ^.^ The EODs of all gymnotiforms are used for object location in the dark and communication, but *Electrophorus* generates three types of EODs: a low-voltage (~10V in adults) discharge for electrolocation and communication emitted by Hunter's organ, an intermediate voltage (~30V) discharge of unknown function produced by the Sachs organ^7^, and high-voltage (typically >300V in adults) discharges for defense and predation produced by the main organ^6^ ^,^ ^8^ ^,^ ^9^ (Figure 1).

Electric eels are common throughout northern South America, particularly in the Amazon, but are absent from large river channels^5^. Human settlements are often located along streams (igarapés) and seasonally flooded areas, which are extensively used for fishing, bathing, and other activities, putting eels and people into frequent contact^4^ ^,^ ^5^.

In cases involving putative death by electric eels, to date, no cutaneous or systemic necrological signs have been observed that would unequivocally implicate electric eels, while also rejecting more mundane possibilities, such as drowning^4^. Consequently, it is impossible to assess whether electric eels constitute a major public health problem, and data are unavailable to assist government agencies with preventative measures such as identifying locations with a higher probability of accidents. Here, we address this shortfall by documenting two clinical cases involving electric eels, one of which resulted in fatality. Our report also presents the first evidence of an accident involving electric eels that could be measured by *in vivo* or *postmortem* hematological examinations.

## CASE REPORTS

**FIGURE 2:**
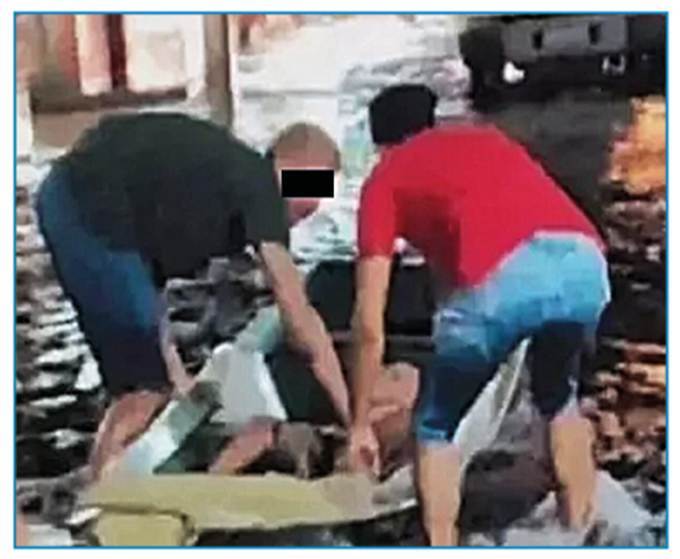
The victim (Case 2) being transported to the hospital in a small boat by family and friends, from G1 News Portal (https://g1.globo.com/ap/amapa/noticia/2022/05/03/jovem-fica-com-lado-esquerdo-do-corpo-paralisado-apos-ataque-de-peixe-eletrico-no-amapa.ghtml.).

**Case 1:** An 18-year-old man was admitted to the Hospital of Laranjal do Jari, Amapá State, Brazil, after family members and others witnessed him suffering a shock from an electric eel. The incident occurred on the streets of his urban house, which had been flooded by the Jari River. The patient reported tremors and was unable to move. The patient was quickly rescued and transported to the hospital by boat (Figure 2). 

General life support measures were administered at the time of care and his movements returned; however, the patient continued to present with muscle contractions and paresis of the lower limbs. Troponin test results were non-reactive, but the levels of creatine phosphokinase (CPK) and creatine phosphokinase myocardial band (CPK-MB) were relatively high (619 u/L and 90 u/L, respectively), consistent with rhabdomyolysis following potent electric discharges. The lower limb paresis and contractions were consistent with rhabdomyolysis and muscle weakening. Muscle injury caused by electrical shock can result in hyperkalemia, hyperphosphatemia, and myoglobinuria. The eel involved in the incident was not recovered; however, given that in the geographic location, both *E. voltai* and *E. varii* occurred, but *E. varii* was more abundant in the anthropized areas where the accident occurred, it was more likely that it was *E. varii*.

**FIGURE 3:**
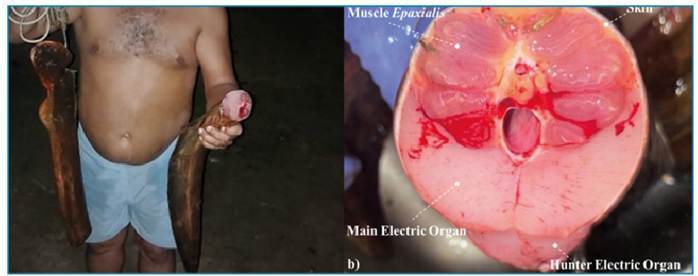
The electric eel involved in Case 2, collected and killed **(a)** with its main electric organ **(b)**, the primary source of the high-voltage electric organ discharges, exposed.

**Case 2:** In September 1997, a doctor on duty in an emergency service at a riverside hospital in Northern Tocantins State, Brazil, responded to an emergency with a 23-year-old male patient whose companions reported seeing him come into contact with an electric eel. The incident occurred in a shallow section of a small tributary of the Tocantins river where they were bathing. The patient's companions reported that he convulsed at the surface of the water, then submerged and disappeared from sight. He was found approximately half an hour later and transported to the Emergency Room by boat (Figure 2), where he was declared dead on arrival. There were no external signs of trauma, and an examination revealed the presence of foam in the airways, consistent with drowning. Remarkably, the eel was captured by the victim’s companions soon after the incident (Figure 3). The image quality in Figure 3 is insufficient to facilitate species-level identification; however, given its geographical locality, *E. voltai* is the most likely species.

## DISCUSSION

We preface our analysis with a brief review of how electric eels deploy high-voltage defensive shocks that pose a threat to humans. Electric eel ‘shocks’ comprise volleys of their high-voltage EODs (derived from the main EO), each comprising from tens to hundreds of individual monophasic EODs (containing only DC content) and lasting from ~0.1 to 20 seconds long^10^. When used defensively, several such volleys are produced in rapid succession. These volleys induce muscular paralysis by activating the motor nerves of the target animal and forcing its skeletal muscles to contract involuntarily^8^. The effect is to immobilize prey animals long enough to prevent an escape response, and the volleys are often repeated during the manipulation of the prey in the mouth of the electric eel prior to swallowing, with a new burst every time the eel releases the jaw grip on the prey^8^. In defensive situations, the electric eel produces repeated volleys and can ‘climb’ up the target animal, partially out of the water, to maximize the immobilization effect^8^. Remarkably, the pulse frequency during these volleys (~300-500 Hz) is evolutionarily optimized to maximize the ability to cause muscular contractions^8^. 

Because the pulses are short (~1-2 ms) and separated by longer interpulse intervals (~2-3 ms), electric eel volleys do not cause significant heating of the target animal’s tissues and, therefore, cannot cause burns. The lack of heating explains why the circuitry of ‘electric fish finders’ used by researchers to detect electric fishes, can utilize resistors and capacitors rated for very low voltages and currents and yet remain undamaged by severe and prolonged close-range electric eel shocks^11^. Given these considerations, the defensive shocks of electric eels are predicted to cause severe muscular contractions, posing a risk of drowning and potentially leading to rhabdomyolysis, but will not cause burn-like lesions. 

The paresis or partial loss of motor function in the lower limbs of the patient in Case 1 may have been caused by muscle injuries resulting from violent and repeated muscular hypertension of electric eels routinely induced in their prey. Clinical manifestations included rhabdomyolysis, as indicated by the measured levels of CPK and CPK-MB^12^. We are unaware of other potential causes of the rhabdomyolysis observed in this incident and conclude that this case represents the first recorded clinical evidence of severe injury involving electric eels. Myological damage is likely related to muscular hypertension induced by strong electrical discharges from electric eels. 

In Case 2, we suspected that the cause of the mortis was drowning, with the victim rapidly immobilized by muscular tetanus induced by repeated shocks from the electric eel. The observation of convulsions and struggles in the victim following contact with the eel, before he was submerged, are all consistent with studies of electric eels defensively shocking potential predators^8^. The presence of foam in the victim’s mouth was consistent with death due to drowning^3^. Unfortunately, there was no information on the CPK and CPK-BM values of the victim in Case 2.

Both the cases reported here present some commonalities with other reported accidents involving electric eels^5^. Accidents with electric eels are often reported to involve groups of partially submerged people engaged in recreational aquatic activities, such as swimming or playing *-* matching the scenarios in Cases 1 and 2. In addition, where fatalities involving electric eels have been previously reported, death is not reported to occur immediately, but instead after a period of intense struggle, as reported in Case 2. It is important to emphasize that the accidents involving both humans and electric eels described in this study occurred with a single touch on the animals. Nonetheless, while the cases we documented here were accompanied by reports from medical professionals, reports of accidents involving electric eels are generally lacking in detailed medical documentation^5^. 

To our knowledge, the rhabdomyolysis documented here is the first clinical injury related to contact with electric eels. We also present the first reliable clinical report of death caused by an electric eel, with the cause of mortis likely to be drowning. In both cases, there were no external lesions or marks, and no evidence of burns. In accidents involving electric eels, we recommend in vivo or post-mortem hematological examination to measure cardiac enzymes, as well as the measurement of other clinical data related to rhabdomyolysis, such as blood potassium and phosphorus levels and urine myoglobin levels. These tests may accurately diagnose the cause of death or serious injury in such cases. For suspected electric eel shock, we recommend appropriate monitoring of renal complications (in such cases, hospitalization may be required) and subsequent application of analgesics and intravenous hydration.. Accidents may be prevented by posting signs in public bathing areas where electric eels frequent, and by consulting with local communities before engaging in recreational and fishing activities in eel-inhabited watercourses.
